# Phospholipid Scramblases: Role in Cancer Progression and Anticancer Therapeutics

**DOI:** 10.3389/fgene.2022.875894

**Published:** 2022-03-29

**Authors:** Himadri Gourav Behuria, Sabyasachi Dash, Santosh Kumar Sahu

**Affiliations:** ^1^ Laboratory of Molecular Membrane Biology, Department of Biotechnology, Maharaja Sriram Chandra Bhanjadeo University, Baripada, India; ^2^ Department of Pathology and Laboratory Medicine, Weill Cornell Medicine, New York, NY, United States

**Keywords:** scramblase, PLSCR, tumor microenvironment, cell signaling, TMEM16F, sheddase activity

## Abstract

Phospholipid scramblases (PLSCRs) that catalyze rapid mixing of plasma membrane lipids result in surface exposure of phosphatidyl serine (PS), a lipid normally residing to the inner plasma membrane leaflet. PS exposure provides a chemotactic eat-me signal for phagocytes resulting in non-inflammatory clearance of apoptotic cells by efferocytosis. However, metastatic tumor cells escape efferocytosis through alteration of tumor microenvironment and apoptotic signaling. Tumor cells exhibit altered membrane features, high constitutive PS exposure, low drug permeability and increased multidrug resistance through clonal evolution. PLSCRs are transcriptionally up-regulated in tumor cells leading to plasma membrane remodeling and aberrant PS exposure on cell surface. In addition, PLSCRs interact with multiple cellular components to modulate cancer progression and survival. While PLSCRs and PS exposed on tumor cells are novel drug targets, many exogenous molecules that catalyze lipid scrambling on tumor plasma membrane are potent anticancer therapeutic molecules. In this review, we provide a comprehensive analysis of scramblase mediated signaling events, membrane alteration specific to tumor development and possible therapeutic implications of scramblases and PS exposure.

## Introduction

Despite favorable advancement in diagnostic and therapeutic strategies, generalized state of the art treatment options for cancer is still lacking. This therapeutic pitfall in cancer treatment is mostly due to the highly intricate and diverse mechanism of cancer development, progression, clonal evolution and drug resistance (Mansoori et al., 2017; Williams et al., 2019). Compared to previous healthcare standards such as chemotherapy, radiotherapy and surgery, immunotherapy has significantly enhanced the cancer care as mainstream, adjuvants and neoadjuvant therapeutics (Esfahani et al., 2020). Tumor cells establish their tissue-specific tumor microenvironment (TME) that enables tumor specific signaling events for tumor survival, drug resistance and metastasis. In addition to expression of many oncogenic proteins on plasma membrane, tumor cells secrete many signaling factors into the exracellular milieu that triggers drug resistance, tumorogenesis, cell migration and adhesion. Malignant cells reorganize their plasma membrane lipids and secrete microvesicles that play a significant role in TME signaling (Jurj et al., 2020).

With their unique repertoire of lipids and proteins, plasma membranes of cancer cells are proficient biosensors of cellular vicinity. In quiescent cells, plasma membrane is polarized with asymmetric organization of membrane lipids across cytosolic and exoplasmic leaflets. Amino phospholipids (PLs) such as phosphatidyl ethanolamine (PE) and phosphatidyl serine (PS) are predominantly localized in the cytosolic leaflet, whereas, phosphatidyl choline (PC) and sphingomyelin (SM) are predominantly localized in the exoplasmic leaflet. This membrane asymmetry is acutely regulated by a set of lipid translocators named flippases and scramblases. Flippases such as aminophospholipid translocase (APTs) facilitate ATP dependent vectorial translocation of PE and PS from exoplasmic leaflet to the cytosolic leaflet. However, scramblases facilitate ATP-independent, random shuffling (scrambling) of membrane lipids in activated cells (Daleke, 2003; Sahu et al., 2007; Contreas et al., 2010). In activated cells, collapse of plasma membrane asymmetry is facilitated by inhibition of ATP-dependent flippases and activation of Ca^2+^/caspase-dependent scramblases (Zalba, and Ten Hagen, 2017). Scrambled plasma membranes are observed in many pathological conditions such as ischemia, hematological disorders, infection and malignancy (Zwaal et al., 2005; Barber et al., 2009; Slone et al., 2015). Multiple proteins such as PLSCRs, TMEM16 family members and Xkr are established scramblases that exhibit rapid mixing of plasma membrane lipids. Scrambled plasma membranes are characterized by the surface exposure of PS that is detected by FITC-labeled annexin binding assay. Surface exposed PS activates membrane bound cellular processes such as apoptosis, blood coagulation, exocytosis, viral entry, innate immunity and inflammatory responses (Kay and Fairn, 2019; Nagata et al., 2020). In addition, scramblases facilitate secretion of cellular components and shedding of microvesicles for paracrine signaling.

PS exposure was classically thought to be an eat-me signal for macrophages displayed on the surface of apopotic cells that promotes their non-inflammatory clearance (efferocytosis). However, in tumors, PS exposure triggers immunosuppressive signaling events leading to attenuation of dendritic cell (DC), natural killer (NK) cell, activation and conversion of tumor-associated macrophages (TAMs) into anti-inflammatory or M2 macrophages. Although the detailed mechanism remains unclear, most solid tumors and tumorigenic cell lines exhibit high constitutive PS exposure. Cell lines B16F10, SH5YSY, C6, HCT116, MOLT4, MOLT3, SW480, U937, EAC, MCF7, AGS exhibit >15% PS exposure on cell surface (De et al., 2018). Unlike apoptotic PS exposure, non-apoptotic PS exposure on malignant cells appears to be reversible, caspase 3/7-independent, Ca^2+^-dependent and exhibits a much slower kinetics (in days) (Birge et al., 2016). Further, the spatiotemporal organization and density of PS exposure on non-apoptotic cell surface differs from the apoptotic cells that enables phagocytes to distinguish both cell types. High PS exposure in cancer cells is triggered by inhibition of ATP-dependent flippases, and activation of scramblases, increased oxidative stress, increased lipid synthesis and altered TME (Koundouros and Poulogiannis, 2020). A scrambled PM with high PS exposure in cancer cells provides an immunosuppressive signal for immunocytes in TME that facilitates tumor survival, progression and metastasis (Birge et al., 2016).

**FIGURE 1:**
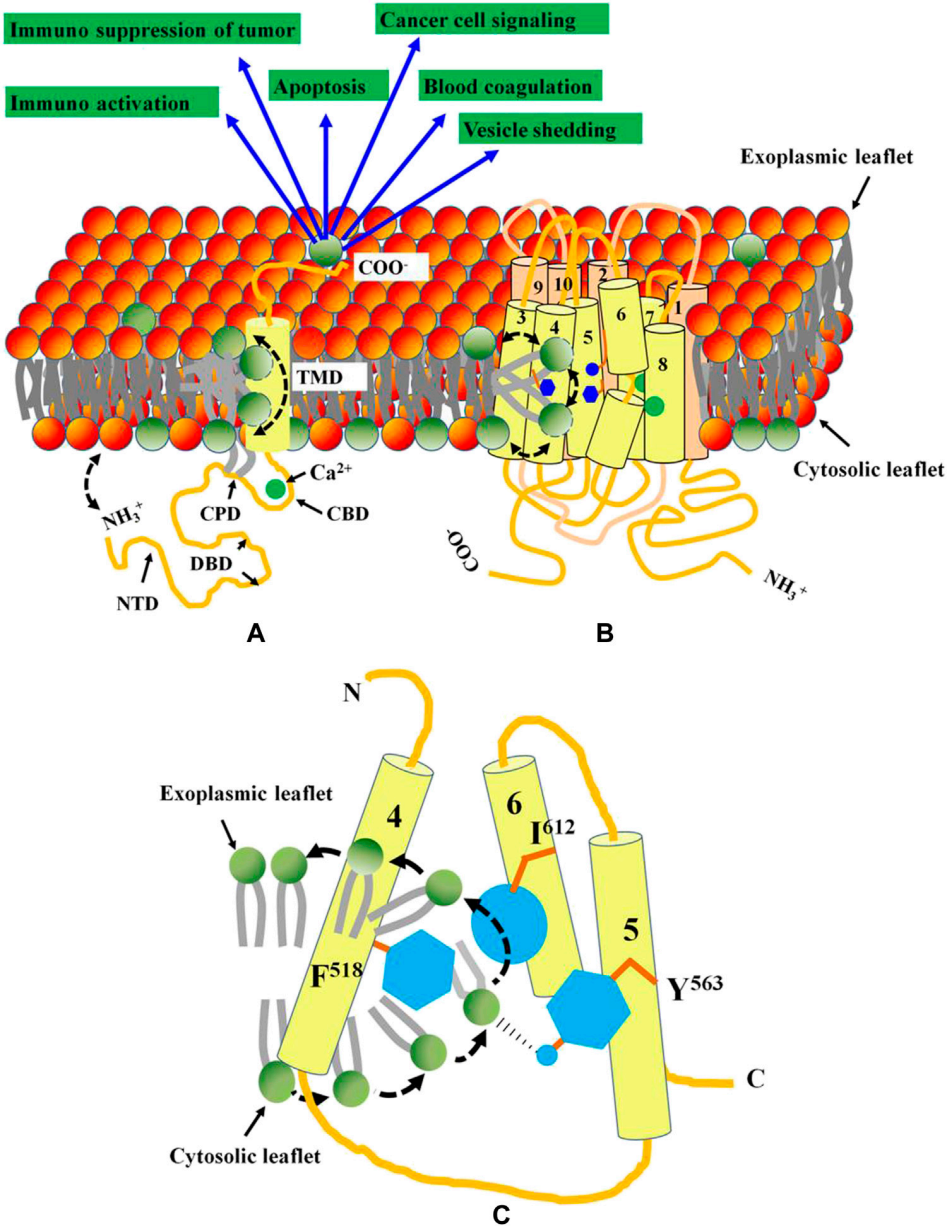
Functional domains and topology of PLSCR1 and TMEM16F. **(A)** PLSCR1 is a type II membrane protein with a short exoplasmic C-terminal tail, a transmembrane domain, a Ca2+-binding EF-hand like domain, a nuclear localization signal, a cysteine palmitoylation domain and a cytosolic N-terminal domain. Ca2+ binding to the EF-hand like domain, plausibly leads to conformational changes in the N-terminal domain essential for PL scrambling. The bilayer defect created by the close proximity of NTD towards plasma membrane results in transbilayer scrambling of lipids. **(B)** The TMEM16F has 10 transmembrane domains with both N and C-termini localized to cytosol. The Ca^2+^-binding domains are localized at the interface between TMDs 6, 7 and 8. A hydrophilic groove created by TMDs 4, 5 and 6 facilitates PL scrambling. **(C)** The PL scrambling pathway is gated by three residues (shown in blue) F^518^, Y^563^ and I^613^ on TMDs 4, 5 and 6 respectively. While the non-polar side chains of F^518^ and I^613^ facilitate the stabilization of the acyl chains of PL, the–OH in polar Y^563^ facilitates the flipping of the head group. Activation of both PLSCR1 and TMEM16F leads to PS exposure on cell surface that activates multiple cellular events.

To date, multiple membrane proteins such as PLSCRs, TMEM16F, Xkr8 and GPCR family members are proposed to scramble PM lipids. Ironically these proteins are of different origin and exhibit little homology among themselves (Stanfield and Horvitz, 2000; Suzuki et al., 2010; Goren et al., 2014; Suzuki et al., 2014). Unlike other scramblases, PLSCRs and TMEM16 family members exhibit Ca^2+^-induced non-apoptotic exposure of PS on different cancer cells. PLSCR1 is a 318 aa long, multifunctional type II membrane protein that contains an N-terminal domain (NTD) (Met^1^-Trp^85^), a DNA-binding domain (DBD) (Met^86^-Glu^118^), a cysteine palmitoylation domain (CPD) (Cys^184^-C^189^), a nuclear localization signal (NLS) (Gly^257^-Ile^266^), an EF-hand like Ca^2+^-binding domain (CBD) (Asp^237^-Asp^284^), a transmembrane domain (TMD) [Lys^288^-Glu^306^] and a short exoplasmic C-terminal tail. Ca^2+^ induced conformational change in PLSCR1 leading to the proximity of the N-terminal domain towards the plasma membrane introduces bilayer defects that stimulates PL scrambling (Figure 1A). However, in TMEM16F, a Ca^2+^ induced conformational alteration regulates the PL scrambling gateway lined by Phe^512^-Tyr^563^-I^612^. Whereas, the hydrophobic side chains of Phe^512^ and I^612^ stabilize the non-polar tails, the polar hydroxyl group of Tyr563 facilitates flipping of the head group (Figures 1B,C). PL scrambling leads to surface exposure of PS, that in turn, activates multiple cellular events such as apoptosis, blood coagulation, tumor cell signaling and immunoactivation.

The cancer genome atlas (TCGA) analysis revels that PLSCR1 is oncogenic and highly over expressed in ovarian carcinoma (Kodigepalli et al., 2013). However, studies on different cancer types have revealed controversial roles of PLSCR1 and their complex regulatory mechanism leading to malignancy (Kodigepalli et al., 2015). Recent investigation has revealed multiple roles of TMEM16 family members in cancer pathogenesis, progression and metastasis (Jacobsen et al., 2013). In addition, many synthetic molecules such as multi-stranded DNAs, peptides and phytochemicals are able to induce membrane lipid scrambling (Ohmann et al., 2018; Dietel et al., 2020; Behuria et al., 2021). This review is aimed at the recent advances on the role of lipid scramblases in cancer cell signaling and anticancer therapeutic strategies.

## Role of Ca^2+^-Activated Phospholipid Scramblases in Cancer Cell Signaling

### PLSCRs

PLSCRs are type II single pass transmembrane proteins initially identified as Ca^2+^-activated PL scramblases. Three other homologs hPLSCR2, hPLSCR3 and hPLSCR4 that exhibited 59, 47 and 46% sequence homology to hPLSCR1 respectively (Wiedmer et al., 2000). PLSCR1 is 37 kDa protein localizes either to PM or nucleus depending upon its state of palmitoylation (Wiedmer et al., 2003). PLSCR3 is localized exclusively to mitochondria and regulates its structure, function and apoptotic responses (Liu et al., 2003). Both PLSCR2 and PLSCR4 are localized to the nucleus and are involved in cell signaling mechanisms (Wiedmer et al., 2000; Sims et al., 2001). Subsequent finding revealed that PLSCRs are multi-functional proteins exhibiting both scrambling and non-scrambling activities. PLSCR1-knockout mice possessed normal scramblase activity, rather exhibited defective hematopoesis (Zhou et al., 2002). Scott syndrome patients, despite being deficient in platelet-scramblase activity, exhibited normal level of PLSCR1 expression in B lymphocytes (Zhou et al., 1998), suggesting the presence of alternative additional scramblases. Investigation revealed that TMEM16F (Ano6) a Ca^2+^-activated Cl^−^ channel that also exhibits PL scrambling in plasma membrane exhibits a homozygous null mutation in Scott Syndrome patients (Nagata et al., 2016). PLSCR1 enhanced granulocyte production in response to growth factors and interferon (IFNs) (Der et al., 1998; Zhou et al., 2002; Dong et al., 2004). These findings revealed PLSCR1 to be a novel signaling protein regulating cellular differentiation and apoptotic responses. Subsequent findings revealed that PLSCRs are onco-regulatory proteins associated with many cancer types such as leukemia and carcinomas.

#### Leukemia

PLSCR1 regulates hematopoesis and glanulopoesis of blood cells. Both *in vivo* and *in vitro* studies show over expression of PLSCR1 in multiple mouse and human leukemic cells (Nakamaki et al., 2002; Yokoyama et al., 2004). Mouse leukemic cells (e.g., U937, NB4 and HT93) over express both normal (MmTRA1a) and truncated (MmTRA1b) isoforms of PLSCR1, previously known as monocytic cell derived transplantability-associated gene 1 [*MmTRA1*] (Kasukabe et al., 1998). Over expression of PLSCR1 is used as a prognostic marker for acute myelogenous leukemia (AML) (Yokoyama et al., 2004). PLSCR1 is over expressed during all-trans-retinoic acid and Methylthioamphetamine-induced differentiation of mouse and human leukemic cells (e.g., HL-60) into granulocytes. Suppression of PLSCR1 expression significantly inhibited the differentiation process (Nakamaki et al., 2002; Wu et al., 2015).

PLSCR1 is essential for differentiation of blood cell, as *PLSCR1* ^−/−^ mice exhibited defective hematopoesis (Zhou et al., 2005). Granulocyte stimulating factor (GCSF)-induced granulopoesis of myelocytes is mediated by PLSCR1 that prolongs mitotic expansion of granulocyte precursors (Chen et al., 2011). PLSCR1 stimulates granulocyte-like differentiation of myeloid leukemic cells with enhanced sensitivity to etoposide-induced apoptosis (Huang et al., 2006). PLSCR1-induced leukemic cell differentiation is mediated by phosphorylation of PKCδ. ATRA and PMA-induced over expression of PLSCR1 in leukemic cells is accompanied by phosphorylation of PKCδ. Rottlerin, an inhibitor of PKCδ-phosphorylation inhibits the differentiation process. Constitutive expression of PKCδ enhances phosphorylation of PLSCR1 and stimulates leukemic cell differentiation (Zhao et al., 2004). In U937 myeloid leukemia cells, PLSCR1 significantly decreases the proto-oncogene c-Myc and antiapoptotic Bcl-2 leading to suppression of leukemia (Huang et al., 2006).

PLSCR1 negatively regulates the protective autophagy in mantle cell lymphoma (MCL) induced by 9-cis-retinoic acid (RA)/Interferon (IFN)-α (RA/IFNα). PLSCR1 is up regulated in response to anticancer drugs such as doxorubicin or bortezomib when used in combination with RA/IFN-α. Upregulation of PLSCR1 inhibits formation of the autophagosome forming complex (ATG12/ATG5/ATG16L1) by binding to ATG12/ATG5 (Mastorci et al., 2016). Treatment of NB4 leukemic cells with the anticancer drug sodium selenite increases the expression of PLSCR1 that induces apoptosis and inhibits protective autophagy in these cells (Shi et al., 2020). PLSCR1 expression level correlates with improved prognosis in acute myelogenous leukemia patients (Li H. et al., 2017).

#### Colorectal Carcinoma

PLSCR1 is over expressed in human colorectal cancer (CRC) cells and hepatic cancer cells. Presence of PLSCR1 in PM-derived microvesicles of CRC patients is a diagnostic marker of colon cancer (Mathivanan et al., 2010; Kuo et al., 2011). PLSCR1 interacts with Fas-ligand that is related to the hepatic metastasis of colorectal carcinoma (Fy et al., 2005). The N-terminal domain of PLSCR1 is known to play a significant role in regulation of CRC malignancy and metastasis. Antibody against N-terminal domain of PLSCR1 (NP1) decreases adhesion, growth, proliferation and metastasis. In addition, PLSCR1 blockade induces intrinsic apoptotic pathway in CRC cells by activating caspases 8, 9 and 3 (Fan et al., 2012; Chen et al., 2014). NP1 treatment significantly decreased the expression of cyclin D1 leading to cell cycle arrest at G1/S phase. NP1 decreased phosphorylation level of signaling proteins Src, Shc and Erks, indicating inhibition of EGF-induced growth signaling cascade mediated by PLSCR1 (Sun et al., 2002; Nanjundan et al., 2003).

#### Hepato-Pancreatic Carcinoma

PLSCR1 is over expressed in metastatic hepatic carcinoma. Silencing PLSCR1 using siRNA in hepatic cancer (LoVo) cells inhibit their proliferation, adhesion, migration and invasion suggesting PLSCR1 to be a key regulatory protein that contributes to the malignancy of liver cancer cells (Cui et al., 2012). Midkine, a heparin-binding growth factor that plays pivotal role in tumorigenesis and tumor progression is localized to nucleus and interacts with PLSCR1. This interaction is proposed to be essential for tumor survival and progression (Yan, et al., 2012; Huang et al., 2015). PLSCR1 is oncogenic in pancreatic ductal adenocarcinoma (PDAC) cells. The anticancer microRNA miR-628-5p accelerates tumor suppression in human PDAC, probably by directly targeting the PLSCR1 and insulin receptor substrate 1 (IRS1) genes through inhibition of AKT/NF-κB signal pathways (Zhou et al., 2020).

#### Ovarian Cancer

PLSCR1 exhibits anti-proliferative and anti-tumor activity on human ovarian epithelial cancer cells. Over expression of PLSCR1 suppress growth of malignant tumor in athymic nude mice. Tumor from PLSCR1-transfected cancer cells were greatly reduced in size, showed increased infiltration of leukocytes and macrophages and differentiated into spindle shaped morphology (Silverman et al., 2002). The mechanism of PLSCR1-mediated suppression of ovarian tumor presently remains elusive. However, PLSCR1 regulates SnoN/SkiL-dependent pathway in ovarian cancer cells. Interferon (IFN-2α) and anti-cancer drug As_2_O_3_ modulate level of PLSCR1 in ovarian carcinoma cells (Kodigepalli et al., 2013). Transfection of normal ovarian epithelial cells with ds DNA activates STING/IRF3 pathway through of induction of PLSCR1 and TLR. Interestingly, this activation pathway is absent from ovarian cancer cells, suggesting its dysregulation in malignant ovarian cells (Kodigepalli and Nanjundan, 2015).

#### Breast Cancer

Expression and Tyr-phosphorylation of PLSCR1 is increased in basal-like breast cancer (BLBC) cells that facilitates its nuclear translocation. PLSCR1 in turn, enhances transactivation of STAT1 through STAT3 upregulation that helps breast tumor survival, metastasis and drug resistance (Huang et al., 2020). A recent study showed that STAT1 promoted breast cancer progression by increasing its cancer stem cell CSC properties (Qadir et al., 2017). This finding shows PLSCR1 to be a novel regulator of breast cancer progression.

### TMEM16 Family Members (Anoctamins)

The TMEM16 (anoctamin) family has 10 isoforms named ANO1 (TMEM16A) to ANO10 (TMEM16K) that were initially discovered as Ca^2+^-activated Cl^−^ ion channels. Subsequent analysis revealed that five members of anoctamin family: TMEM16C (ANO3), TMEM16D (ANO4), TMEM16F (ANO6), TMEM16G (ANO7) and TMEM16J (ANO9) exhibit Ca^2+^-dependent PL scrambling activity in addition to their role as Cl^−^ channel (Suzuki et al., 2013). However, both PL scrambling and Cl^−^ channel activities are independent of each other. In particular, TMEM16F (ANO6) is the major contributor to the process of PS translocation from the inner to the outer leaflet of the plasma membrane (Pedemonte and Galietta, 2014). Different paralogs of anoctamins are expressed during murine embryogenesis, indicating their role in embryonic development and cellular differentiation (Rock and Harfe, 2008). Growing set of evidences suggest that the members of TMEM16 family are over expressed in cancer cells that is associated with poor prognosis and cancer development (Katoh and Katoh, 2003).

#### TMEM16A

Most studies on the role of TMEM16 family members in cancer were performed on TMEM16A that doesn’t exhibit PL scrambling activity. TMEM16A is over expressed in head and neck squamous cell carcinoma, gastric and colorectal cancer that enhances cancer cell survival, proliferation, migration and metastasis. It’s inhibition led to suppression of cancer cell proliferation and migration (Kalienkova et al., 2021).

#### TMEM16F (ANO6)

TMEM16F is associated with cell shrinkage, migration and invasion. TMEM16F is highly expressed in myoblasts and regulates it’s proliferation and differentiation via the ERK/AKT signaling pathway (Zhao et al., 2014). In breast cancer malignancy, the splicing pattern of TMEM16F mRNA regulates the metastatic potential. Analysis of murine breast tumor transcriptomes reveals alternative exons splicing of TMEM16F mRNA that influences the metastatic capacity and poor prognosis of mammary cancers (Dutertre et al., 2010).

#### TMEM16G (ANO7)

TMEM16G is exclusively over expressed in prostrate cancer cells, making it a novel prognostic marker and immunotherapeutic drug target (Bera et al., 2002). TMEM16G and it’s two single nucleotide polymorphic forms are linked to the aggressiveness and progression of prostrate cancer (Kaikkonen et al., 2018). The long spliced form of TMEM16G is concentrated at the cell-cell contact sites of plasma membrane that might play a vital role in regulation of adhesion in human prostate adenocarcinoma cells (Das et al., 2007). TMEM16G interacts with AP2B1, COPG2, HSPA1A and SND, the proteins that are involved in vesicle maturation and trafficking. COPG2 and AP2B1 participate in vesicle maturation and trafficking. HSPA1A and SND localize to the plasma membrane that regulates MV shedding into the TME (Kaikkonen et al., 2018). HSPA1A and SND expression regulate cancer aggressiveness and resistance to anticancer therapeutics (Blanco et al., 2011; Shevtsov et al., 2018).

#### TMEM16J (ANO9)

Up regulation of TMEM16J expression is linked to progression and metastasis of stage II and III CRC, indicating it’s role in metastasis and invasion (Li et al., 2015). High expression of TMEM16J is a poor prognostic factor in patients with pancreatic cancer. Exogenous expression of TMEM16J in pancreatic cancer cells *in vitro* and mouse xenograft, significantly increased cell proliferation. it’s knockdown in human pancreatic tumor cells AsPC-1, BxPC-3 and Capan-2 strongly inhibited their proliferation. TMEM16J physically associates with EGFR that underlies TMEM16J-induced cell proliferation as it’s knocking down enhanced the effect of EGFR-targeted anticancer drugs (Jun et al., 2017). Clinical studies on esophageal squamous cell carcinoma (ESCC) reveals that patients with high TMEM16J expression exhibited significantly worst survival, poor prognosis and cell cycle inhibition by reduced expression of centrosome-related genes. siRNA-induced knockdown of TMEM16J in KYSE150 and KYSE790 oesophageal squamous carcinoma resulted in reduced cell proliferation, invasion migration and increased apoptosis (Katsurahara et al., 2020).

## Transcriptional Regulation of Scramblase Expression During Malignancy

Tumor cells exhibit acidic cytosol and poor oxygenation. In addition, the cell cycle is deregulated leading to altered expression of transcription factors. Transcription factors such as c-Myc and snail, drugs such as ATRA and Methylthioamphetamine (MTA), anticancer drugs regulate expression of PLSCRs (Table 1)

**TABLE 1:** Transcriptional regulators of PLSCRs.

Transcriptional Regulator	Cell/Tissue	PLSCR	Regulation	References
Unknown	Leukemic monocytes	PLSCR1	+	Zhao et al. (2005)
Unknown	in hepatic cancer (Lovo) cells	PLSCR1	+	Cui et al. (2012)
III 10	U937 leukemia cells	PLSCR1	+	Qin et al. (2012)
Unknown	Colorectal carcinoma cells	PLSCR1	+	Cui et al. (2012)
IFNα/IFNγ (induced by HCV)	Huh-7 hepatic cancer cell line	PLSCR1	+	Metz et al. (2012)
Woogonoside	U937 leukemia cells and HL-60 cells	PLSCR1	+	Chen et al. (2013)
Unknown	Ovarian carcinoma	PLSCR1	+	Kodigepalli et al. (2013)
SnaiL	IMR-32	PLSCR1	-	Francis et al. (2014)
Unknown	Colorectal carcinoma (stage II and III)	PLSCR1	+	Li et al. (2015)
Histone deacetylase	Head and neck squamous cell carcinoma (HNSCC) cell line	TMEM16A	+	Wanitchakool et al. (2014)
ds DNA	T80 and HEY ovarian cancer cells	PLSCR1	+	Kodigepalli and Najundan, (2015)
miR-424-5p	Non-small cell lung cancer (NSCLC)	PLSCR4	+	Li et al. (2019)
LINC00641 non-coding RNA	Lungs cancer	PLSCR4	+	Li et al. (2019)
IRF3	Ovarian epithelial carcinoma cells	PLSCR1	+	Kodigepalli et al. (2013)
Unknown	Breast epithelium	PLSCR1	+	Huang et al. (2020)
Low pH ([H^+^] > 10^–6^ M)	HEK293 cells	PLSCR1	-	Francis and Gummadi, (2015)
c-Myc	*In vitro* and *In vivo* analysis	PLSCR1	+	Vinnakota and Gummadi, (2016)
ATRA and MAT	NBTZ and CD11b cells	PLSCR1	+	Wu et al. (2015)
9-cis-RA, IFNα, anti-cancer drugs	Cancer cells	PLSCR1	+	Mastorci et al. (2016)
SnaiL	HEK 293, Huh-7 and U-87 MG	PLSCR4	-	Vinnakota and Gummadi, (2016)
Resveratrol	HeLa, cervical cancer cells	PLSCR1	-	Zhao et al. (2019)
P^53^	Selenite treated NB4 leukemic cells	PLSCR1	+	Shi et al. (2020)
Sodium selenite	NB4 leukemic cells	PLSCR1	+	Shi et al. (2020)
STAT 1	Basal like breast cancer	PLSCR1	+	Huang et al. (2020)
Karyopherin α2	Lung adenocarcinoma (ADC)	PLSCR1	+	Liao et al. (2022)
Midkine (MDK)	Hepatocellular carcinoma	PLSCR1	-	Shabgah et al. (2021)
miR-628-5p	Pancreatic carcinoma (PDAC) cells	PLSCR1	-	Zhou et al. (2020)

### c-Myc and Snail Transcription Factors

Both *in silico* and *in vivo* analysis shows that c-Myc is a transcriptional regulator of PLSCR1 with a putative binding site located between −800 and −400 upstream of 5’ the flanking region of *PLSCR1* (Vinnakota and Gummadi, 2016). Both PLSCR1 and PLSCR4 are down regulated at mRNA and protein level by a Snail transcription factor (Snail TF) that binds to the putative promoter regions of PLSCR1 [−1,525 to −1,244] and PLSCR4 [−1,521 to −1,516] respectively (Francis et al., 2014; Vinnakota and Gummadi, 2016). This finding demonstrates key regulatory role of PLSCRs in tumorigenesis.

### Differentiation Inducing Agents

Differentiation inducing chemicals such as Phorbol-12 myristate-13 acetate (PMA), a stimulator of PKCδ is known to enhance transcription of PLSCR1 and stimulates cellular differentiation (Andrews et al., 2002). All-trans retinoic acid (ATRA), elevates PLSCR1 expression in acute promyelocytic leukemic (APL), NB4 and HL60 cells but not in maturation-resistant NB4-LR1 cells (Zhao et al., 2004). ATRA in combination with MTA is shown to be a more robust inducer of PLSCR1 expression that regulates leukemic cell differentiation. In U937 and HT1080 leukemic cells, sequential activation of PKCδ and JNK stimulate phosphorylation of STAT1 at Ser^727^ that in turn activates transcription of PLSCR1 (Zhao et al., 2005).

### Acidic Cytosol and Oxygen Deprivation

Cancer cells exhibit an acidic cytoplasm and tumor cells exhibit an acidic microenvironment. Low pH (<6) leads to transcriptional down regulation of hPLSCR1 that correlates with decreased sensitivity of HEK293 to apoptosis. As the cytosol turns acidic in many malignant cell types, PLSCR1 might be a key regulatory protein of cancer progression (Francis and Gummadi, 2015). Similarly, TME exhibit a low oxygen condition that up regulates PLSCRs. PLSCR1 and 3 are over expressed during oxygen deprivation that negatively regulates cellular stress (Ostojic et al., 2013; Slone et al., 2015).

### Natural and Synthetic Drugs

Sodium selenite enhances apoptosis in NB4 leukemic cells through IRF3-mediated upregulation of PLSCR1 (Shi et al., 2020). III10, a synthetic flavonoid induces differentiation of U937 leukemic cells by enhancing PLSCR1 synthesis (Qin et al., 2012). Paclitaxel, an established anticancer drug increases PS exposure in RBC that leads to blood coagulation and thrombosis. However, if it leads to over expression of any specific scramblase, remains to be determined (Kim et al., 2018). Synthetic enzyme made from eight DNA strands induces scrambling of ∼ 10^7^ lipids in biological membranes. The synthetic DNA scramblase led to rapid PS exposure in human breast cancer cells (MDA-MB-231) (Ohmann et al., 2018). Stimulation of PS exposure in malignant cells using an exogenous scramblase opens a novel anti-cancer therapeutic approach.

Woogonoside, a natural flavonoid from *Panax ginseng* induces differentiation of U937 and HL-60 leukemia cells by upregulating PLSCR1 (Chen et al., 2013). Woogonoside stimulates differentiation of primary AML cells through activation of PLSCR1/IP3R1/Ca^2+^ axis (Li M. et al., 2017). Similarly, resveratol, a polyphenol isolated from grape skin significantly reduces the cervical tumor through over expression of PLSCR1 (Zhao et al., 2019). Many natural anticancer drugs exhibit anticancer activity through over expression of PLSCR1. These compounds are potent drug leads or adjuvant therapeutics that act through inhibition of scramblases or enhance PS exposure on plasma membrane of cancer cells.

## Role of Scramblases in TME Signaling

### Regulation of Tumor Cell Volume, Diapedesis and Migration

Cell shrinkage is an essential morphological feature of metastiatic tumor cells that enables their invasion into the tissue interstitial space (diapedesis) through narrow gaps of capillary endothelial cells. Anoctamins are the key regulators of cellular volume during cell shrinkage. Activation of TMEM16 members is triggered by increased cytosolic Ca^2+^ that activates Cl^−^ channel activity. Cl^−^ export is accompanied by loss of water as a mechanism of osmotic balance that results in shrinkage of the metastatic tumor cells (Sontheimer, 2008; Ruiz et al., 2012). As all the anoctamins exhibit Cl^−^ channel activity, their activation causes simultaneous cell shrinkage and (or) PS exposure on the cell surface. Polarized activation of TMEM16A in migrating cells allows their diapedesis (Sontheimer H. 2008). Change of shape in metastatic carcinoma cells is facilitated by TMEM16A-inducd cytoskeletal remodeling. TMEM16A associates with scaffolding proteins ezrin, radixin, moesin and RhoA that connect plasma membrane proteins to the cytoskeleton (Perez-Cornejo et al., 2012). TMEM16A interacts with various cell adhesion proteins such as zyxin, fibulin 1, S100A11, twinfilin and catenin that falilitates migration and attachment of metastatic tumor cells (Wanitchakool et al., 2014). siRNA-mediated down regulation of TMEM16F was shown to decrease ionophore-mediated release of TNF-Receptor 1 (TNFR1) in human umbilical vein endothelial cells (HUVECs) (Veit et al., 2018). Both TMEM16A and TMEM16F are expressed in Ehrlich Lettre ascites (ELA) cells that regulates cell migration. In metastatic ELA cells, TMEM16A and TMEM16F regulate the direction and rate of migration respectively (Jacobsen et al., 2013). TMEM16F-induced PE exposure on surface of migrating cells is a biomarker of migrating HELA cells which promotes cell migration and adhesion (Fan et al., 2012; Kato et al., 2013).

### Shedding of Microvesicles

Microvesicles (MVs) are small, PM-derived membrane-enclosed entities with diameter 50–200 nm that are shedded by cells of multicellular organisms to transfer cellular components essential for paracrine signaling (Headland et al., 2015; Fujita et al., 2016). Both PLSCRs and TMEM16 regulate shedding of MVs through Ca^2+^-induced PS exposure. PLSCR1 is a positive regulator of MP release as the shedding is greatly reduced in cells deficient in expression of PLSCR1 (Gonzalez et al., 2009). PLSCR1 modulates release of MPs by regulating Ca^2+^ induced K^+^ efflux, PS exposure and enhanced membrane fluidity (Campbell et al., 2014). Both PLSCR1 and 3 are secreted through MVs of colorectal cancer cells that contain signaling machinery for colorectal tumorogenesis (Mathivanan et al., 2010). MPs from both hematological and non-hematological cancer cells carry transcripts of membrane vesiculation machinery, micro RNAs (miRNAs), surface antigens, proteins and genetic materials that re-templates recipient cells so as to reflect traits of the donor cell (Jaiswal et al., 2012).

The long spliced form of TMEM16F is targeted to plasma membrane that regulates PL scrambling, shedding of microvesicles and immune-signaling in TME. TMEM16F regulates the formation of a subset of MVs *via* PL scrambling. TMEM16G peptides were detected in patient-derived prostate-specific MVs (prostasomes) indicating their role in vesicle maturation and trafficking (Poliakov et al., 2009). During prostate cancer progression, prostasomes are secreted into stromal tissue, where they can support tumor growth and induce immune suppression (Lundholm et al., 2014). TMEM16G is immunogenic and TMEM16G-targeted T cells exhibit specificity against prostate cancer cells (Cereda et al., 2010). MVs spread the chemotherapy resistance among cancer cells. For instance, docetaxel chemotherapy resistance between cells spreads via MVs (Corcoran et al., 2012).

### Sheddase Activity of ADAM10 and ADAM17

**FIGURE 2:**
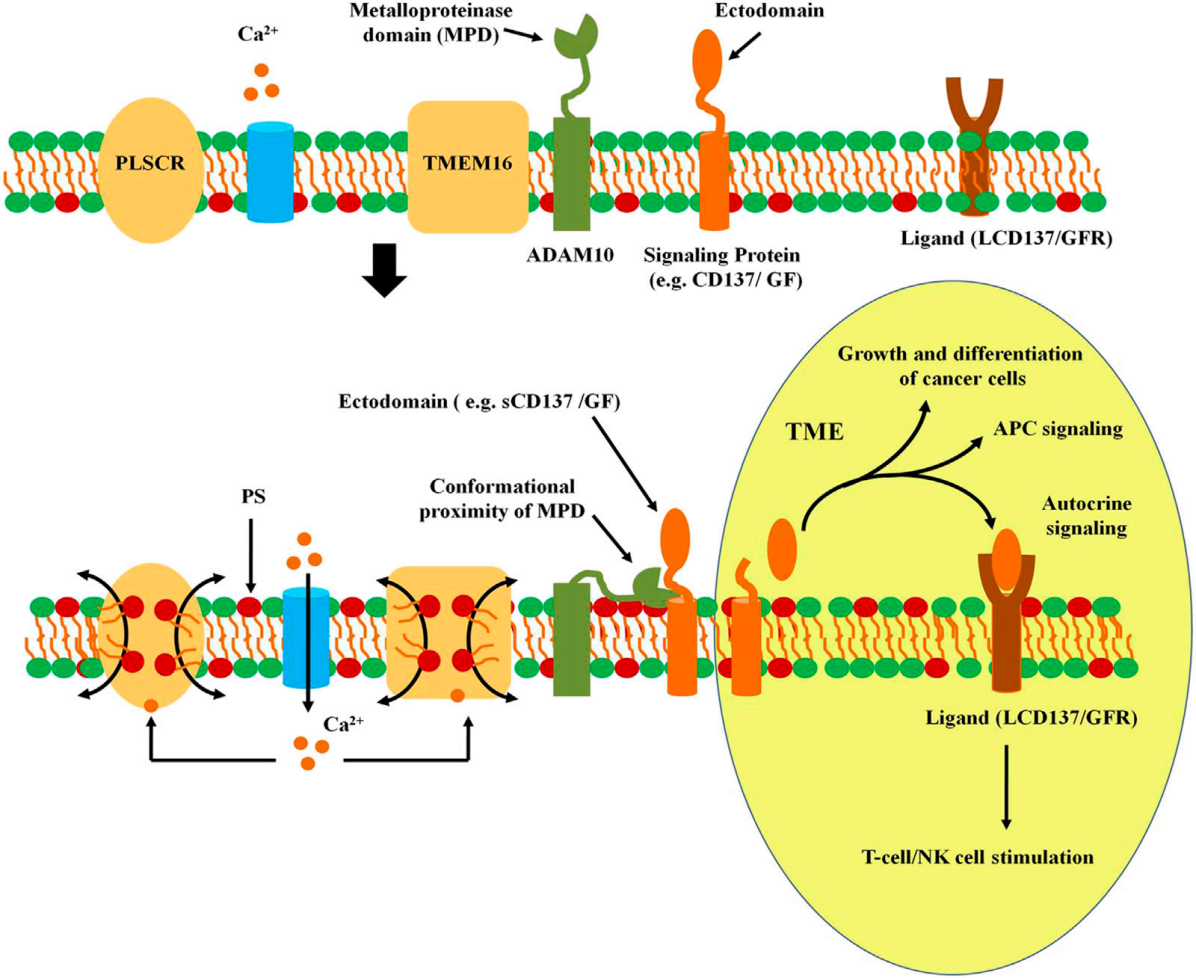
Activation of ADAM sheddase activity. In non-activated quiescent cells, the metalloproteinase domain of ADAM10 remains distant from the signaling membrane protein. However, entry of Ca^2+^ into the cytoplasm activates PLSCRs and TMEM16 scramblases, leading to externalization of phosphatidyl serine. Interaction of the exoplasmic domain of ADAM10 with PS on the surface of plasma membrane leads to conformational alteration in the MPD increasing it’s proximity with the membrane-localized signaling protein. The proteinase acivity in MPD releases the ectodomain that serves as a signal for other tumor cells or immunocytes in the TME. The ectodomain of signaling proteins such as CD137 or membrane anchored growth factor (GF). The ectodomain of CD137 (sCD137) binds to it’s own ligand (LCD137) that leads to autocrine stimulation of T cells or NK cells. Release of GF leads to paracrine signaling in cancer cells leading to tumor growth and metastasis.

Scramblase-mediated PS exposure on surface of cancer cells is an essential cancer regulatory event that facilitates shedding of many membrane-attached proteins to the extracellular medium. The sheddase activity of disintegrin-metalloproteinase ADAM 17 and ADAM10 is regulated by PS exposure on cell surface (Bleibaum et al., 2019) (Figure 2). Ectodomains of transmembrane substrates including TGF-alpha, CD137, Amphiregulin (AREG) and tumor necrosis factor receptor (TNFR) are shed into the extracellular medium by TMEM16F. Proteins such as neuronal (N)-cadherin, epithelial (E)-cadherin, vascular-endothelial (VE)-cadherin, EGFR ligands, *β*-cellulin (BTC), EGF and the low affinity IgE receptor CD23 are secreted into the TME that enhances tumor survival, migration and apoptosis. Expression of constitutively active mutant of TMEM16F that leads to spontaneous PS exposure, led to increased release of AREG and TGF-alpha (Veit et al., 2018). Over expression of TMEM16F increases stimulated shedding of soluble CD137 (sCD137), a member of the TNF receptor family in patients with malignancy. ADAM10 and ADAM17 stimulate shedding of sCD137 and it’s ligand (LCD137) through their interaction with PS exposed on cell surface. A hyperactive ANO6 results in maximal constitutive shedding of CD137 that enhances T cell proliferation in TME (Figure 2). ADAM10 activation could not be induced in lymphocytes of Scott syndrome patients that harbor a missense mutation in TMEM16F. A putative PS-binding motif was identified in the conserved stalk region of ADAM10. Replacement of this motif resulted in strong reduction of sheddase activity (Bleibaum et al., 2019). Targeting of CD137/CD137L signaling pathway in tumor cells by targeting TMEM16F could lead to the development of novel immunotherapeutics against cancer (Seidel et al., 2021). In macrophages TMEM16F is necessary for phagocytosis stimulated by the ATP receptor P2X7 and microglia lacking TMEM16F demonstrate defects in process formation and phagocytosis (Ousingsawat et al., 2015; Batti et al., 2016).

### Immuno-Suppressive Signaling

Tumor survival and immune escape requires IFN-I and II triggered JAK-STAT signaling through expression of IFN-stimulated genes. PLSCRs regulate the INF-mediated immune-signaling leading to the immune escape of tumor cells. The transcriptional activator of IFN-induced genes is the cytosolic ISGF3 complex which is formed by dimerization of either two phosphorylated STATs (STAT1, STAT2 and STAT3) or binding of the dimer with a interferon regulatory factor (e.g. IRF9). PLSCR2 bind to STAT3 that attenuates the adaptive immunity against tumors (Yu et al., 2009; Kuchipudi, 2015). PLSCR1 binds to STAT2 to regulate the promoter activity ISGF3-mediated transcriptional activation (Tsai and Lee, 2018). PLSCR2 binds to the N-terminal domain of STAT3 that inhibits the STAT3-dependent promoter occupancy of ISGF3 complex leading to attenuation of it’s promoter activity (Tsai and Lee, 2018). Palmitoylation of PLSCR2 is essential for it’s STAT3 binding and blockade of the transcriptional activity (Tsai et al., 2019). STAT3 signaling has been widely linked to cancer cell survival, immune suppression and sustained inflammation in the TME. Hence, PLSCRs are the immune regulatory proteins that regulate tumor progression and survival through STAT-regulatory mechanism.

### T Cell Signaling

Ca^2+^ ionophores induce activation of TMEM16F in T cells that stimulates PS exposure that in turn, triggers large-scale cell surface membrane expansion. Continued stimulation of TMEM16F results in shedding of ectosomes that contain T-cell co-receptor PD1. Knocking down of TMEM16F resulted in inhibition of membrane expansion and triggered rapid endocytosis of PD1 upon increased cytosolic Ca^2+^. Presence of TMEM16F prevents PD1 endocytosis by facilitating Ca^2+^-induced lipid scrambling in plasma membrane and activating massive endocytosis (Hilgemann and Fine, 2011). PD-1 is a negative regulator of the immune system that is hijacked by various cancers to evade anti-tumour immune responses. TMEM16F deficiency results in high level expression of PD-1 on cell surface that results in their immune exhaustion (Hu et al., 2016). PD1 is an immune-check point that could be blocked by antibodies which are potent anti-cancer immune therapeutics (Pardoll, 2012). In HEK293T cells, over expression of TMEM16F led to increased Ca^2+^-mediated PS-exposure that was accompanied by enhanced release of amphiregulin (AREG) and TGF-alpha essential for T-cell activation (Figure 2).

### Phytochemicals That Induce Lipid Scrambling in Plasma Membrane of Cancer Cells

Many phytochemicals exhibit anticancer properties through scrambling of plasma membrane lipids in cancer cells. The scrambled plasma membrane that exhibits PS exposure on cancer cell surface results in their apoptotic clearance. However, in solid tumors, the PS exposed on plasma membrane is a novel target for PS-binding anticancer molecules. While many phytochemicals and their synthetic conjugates are increasingly promoted as anti-cancer therapeutics, tumor cells exhibiting high PS exposure on cancer cell surface is immune targeted using high affinity PS-binding agents. Diverse plant compounds such as flavonoids, alkaloids, quinones, terpenoids, polyphenols and tannins exhibit PS exposure in malignant cells (Table 2).

**TABLE 2:** Phytochemicals that induce apoptosis in cancer cells through PS exposure.

Natural molecule	Source	Target cell	Mechanism	References
Genistein	Soy and *T. avellanedae*	Prostate adenocarcinoma (PC3)	Caspase 3 dependent apoptosis	Diaka et al. (2004)
Andrographolide	*A. paniculata*	Hepatocellular carcinoma (HepG2CR)	PS exposure and stimulation of autophagy	Chowdhury et al. (2019)
Woogonoside	*P. ginseng*	Leukemia (U937 and HL-60) cells	Over expression of PLSCR1	Chen et al. (2013)
Resveratol	Grape skin	Cervical tumor	Over expression of PLSCR1	Zhao et al. (2019)
Paclitaxel	*T. brevifolia*	Breast cancer	PS exposure	Kim et al. (2018)
Luteolin	Green pepper	Breast adenocarcinoma (MCF-7/Mito^R^)	Induction of apoptosis	Rao et al. (2012)
Erioquinol	Piper genus	Glioma (U373), Breast cancer (MCF7), lung cancer (A549), prostate cancer (PC-3)	Activation of mitochondrial apoptotic pathway	Munoz et al. (2019)
Bigelovin	*I. helianthus-aquatica*	Colorectal carcinoma (HT-29 and HCT 116)	Increase PS exposure	Li H. et al. (2017)
Sesamol	S. indicum	human lung adenocarcinoma (SK-LU-1)	Induction of caspase (8, 9, and 3/7) mediated apoptosis	Siriwarin and Weerapreeyakul, (2016)
Hispidin	Citrus seeds	Human hepatocellular carcinoma (HepG2)	Down regulation of Bcl-xL and induction of apoptosis by PS exposure	Banjerdpongchai et al. (2016)
Colchicine	*C. autumnae*	PANC-1, BxPC-3 and LeukemicT cells	Nuclear fragmentation, PS exposure	Larocque et al. (2014)
Curcumin	*C. longa*	Breast cancer cells (MDA-MB-231 and MCF-7)	Apoptosis induction by Increased PS exposure	LV et al. (2014)
Apigenin	*P. cripsum*	Bile duct cancer cell (HuCCA-1) Colon cancer (HT-29)	PS exposure	Subhasitanont et al. (2017)

#### Flavonoids

 Treatment of leukemic T-cell with wogonin, a flavonoid, triggers their apoptosis. Wogonin, activates cyclin-dependent kinase 9 (CDK9) leading to down regulation of anti-apoptotic protein myeloid cell leukemia 1 (Mcl-1) in leukemic T cells (Polier et al., 2011). Apigenin, a flavonoid found in *Petroselinum cripsum* stimulates growth inhibition and apoptosis in bile duct tumor (cholangiocarcinoma) cells. Apigenin treatment increases PS exposure by 41% and triggers autophagy in tumor cells (Subhasitanont et al., 2017). Genistein, ([4′,5′,]-trihydroxyisoflavone), from soy has a heterocyclic, diphenolic, structure similar to estrogen. Genistein in combination with *β*-lapachone, a quinone from *Tabebuia avellanedae* having 3,4-dihydro-2H-benzo [h]chromene-5,6-dione structure induced apoptosis in prostate cancer through synergistic activation of caspase 3 and NAD(P)H:quinone oxidoreductase (NQO1) dependent pathways respectively (Diaka et al., 2004). Luteolin, a flavonoid from chamomile tea shows anti-proliferative activity on MCF-7/Mito^R^ multidrug resistant breast cancer cells (Rao et al., 2012). Luteolin that leads to 10% PS exposure in MCF-7/Mito^R^ cells stimulates late apoptosis and necrotic stages of cell death in these cells. Curcumin, a flavonoid from *Curucruma longa* inhibits growth of human breast cancer cells (MDA-MB-231 and MCF-7) through rapid PS exposure and apoptosis stimulation. Curcumin down regulates Bcl-2 in breast cancer cells, while up-regulating Bax, resulting in an increase in ratio of Bax/Bcl-2 (LV et al., 2014). Hispidin, a flavonoid from Citrus seeds induce apoptosis by activating Caspases 9, 8, and 3 in HepG2 cells. Hispidin down regulates the anti-apoptotic protein Bcl-xL and up regulates Bax, Bak, and tBid proteins, activating caspase mediated PS exposure on HepG2 (Banjerdpongchai et al., 2016).

#### Alkaloids

Paclitaxel,an alkaloid from Pacific yew tree (*Taxus brevifolia*) stimulates PS exposure in RBCs that leads to blood coagulation and thrombosis (Kim et al., 2018). Paclitaxel induced 60% increase in apoptosis and G2/M phase cell-cycle arrest in Canine mammary cancer cells by down regulating Bcl-2 and up regulating Bax (Ren et al., 2018). Berberine induced apoptosis in leukemia cells by inhibiting the expression of X-linked inhibitor of apoptosis protein (XIAP) (Liu et al., 2013). Colchicine, an alkaloid from *Colchicum autumnae* exhibits anticancer activity. A synthetic colhicine analogue N-acetyl-O-methylcolchinol induces nuclear fragmentation, PS externalization and cell death in pancreatic epitheloid carcinoma (PANC-1), pancreatic adenocarcinoma (BxPC-3) and acute leukemic T cells (Larocque et al., 2014).

#### Terpenoids

Andrographolide, an anticancer diterpenoid from *Andrographis paniculata* reverses cisplatin-resistance in human hepatocellular carcinoma cells (HepG2CR) through rapid scrambling of plasma membrane lipids, leading to PS exposure (Chowdhury et al., 2019). Diosgenin, an anticancer steroid sapogenin from legumes and yams (e.g., *Dioscorea villosa*) shows significant growth reduction in HepG2 cells. Diosgenin stimulates up regulation of p21, p27, p57 and caspases 3, 7 and 9 proteins (Li et al., 2010). Bigelovin, a sesquiterpene lactone isolated from *Inula helianthus-aquatica* induce caspase 3-mediated apoptosis in human coorectal cancer HT-29 and HCT 116 (Li H. et al., 2017).

#### Polyphenols

Sesamol, a phenolic lignan from sesame seeds (*Sesamum indicum*) induce apoptosis in human lung adenocarcinoma (SK-LU-1) (Siriwarin and Weerapreeyakul, 2016). Sesamol increased the activities of caspase 8, 9, and 3/7, indicating that it enhances apoptotic cell death through both extrinsic and intrinsic pathways. Sesamol led to 34% increase in PS exposure during both early and late stages of apoptosis. Gibbilimbol B, a quinol and eriopodol A, an alkenylphenol from Piper genus enhances cytotoxicity in breast cancer cells by induction of caspase-dependent apoptosis. These molecules suppress the expression of XIAP, an apotosis inhibitor (Munoz et al., 2019).

## Targeting the PS on Malignant Cells for Anti-Cancer Therapeutics

**FIGURE 3:**
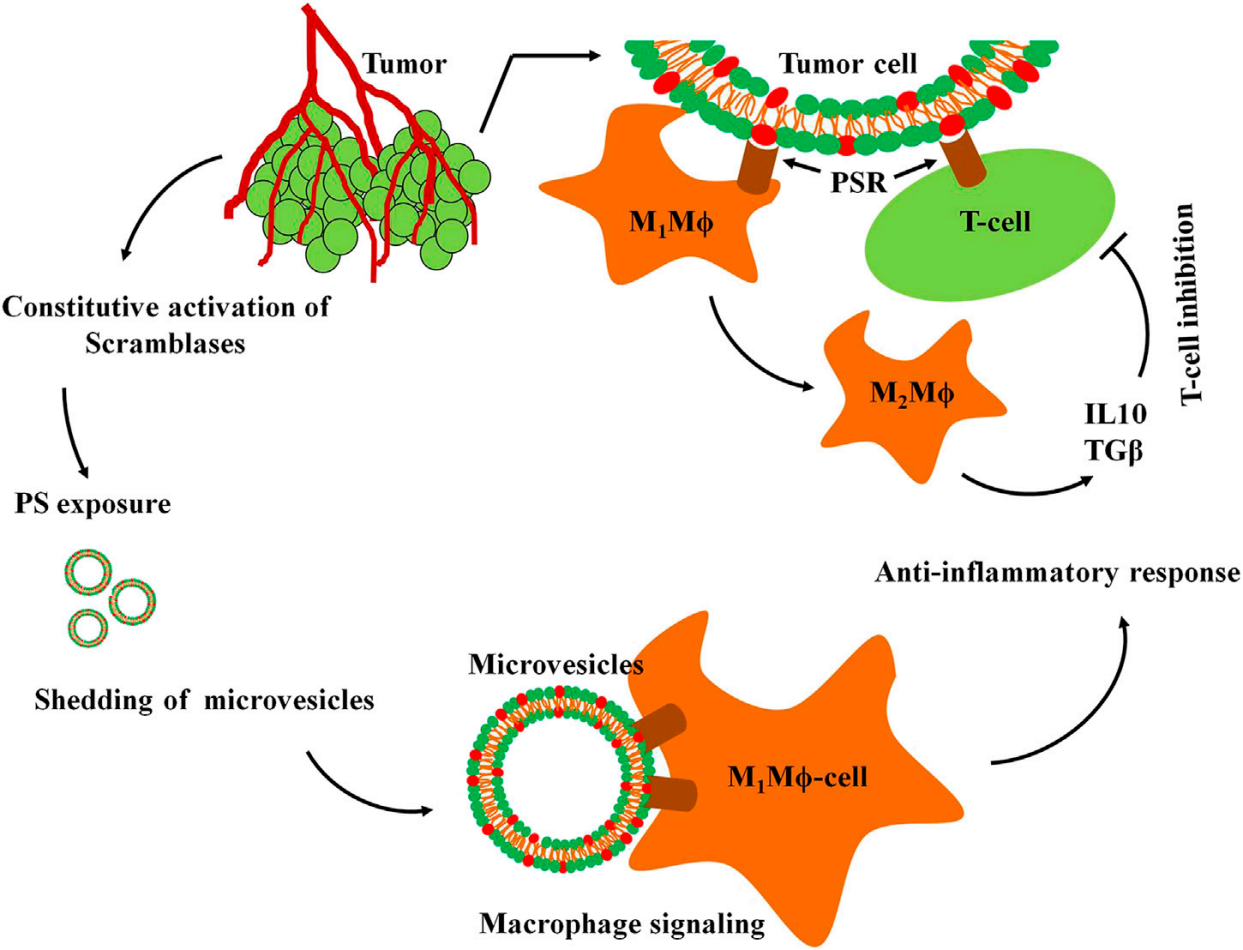
Immunosignaling in the TME. Altered signaling mechanisms in tumor cells activates scramblases resulting in constitutively high PS exposure on their surfaces. PS exposure stimulates shedding of microvesicles (MVs) from tumor cells with surface exposed PS in to the tumor microenvironment. The PS on tumor cells or MVs are recognized by the PS receptors (PSR) of M1Mϕ macrophages and T-cells that initiate the anti-inflammatory response. The inflammatory M1Mϕ macrophages are converted into the non-inflammatory M2Mϕ macrophages that in turn, secrete interleukin 10 (IL10) and tumor growth factor *β* (TGFβ) triggering inhibitory response against T cells.

High constitutive PS exposure on surface of cancer cells is being explored as a novel target for anticancer therapeutics. Structurally and functionally diverse types of molecules with PS-binding affinity are potential targeting agents for solid tumors. Peptides, proteins, synthetic molecules and phytochemicals have been successfully targeted either to the tumor cells or TME (Table 3). High PS exposure in malignant cells exhibit higher uptake of the therapeutics compared to normal cells. PS-binding peptides such as Fc-Syt1, PSBP-6, peptide-peptoid hybrid PPS1 exhibit high affinity towards the negatively charged head group of PS exposed on cell surface and effectively deliver their conjugated therapeutics into the tumor cells. C2A domain of synaptotagmin 1 (Syt-1) that shows high affinity towards phosphatidylserine was fused to the human IgG1-derived Fc fragment to form the fusion protein Fc-Syt1. The fusion protein was in turn conjugated to cytotoxic drug monomethyl auristatin E. This protein-drug conjugate (PDC) effectively targeted the drugs into the human breast cancer cells MDA-MBA-231 (2H11) in mouse xenograft models (Li M. et al., 2017). Annexin V binds to PS exposed on surface of HUVEC cells to enhance their apoptosis in murine melanoma xenograft model. Treatment with Annexin V showed significant reduction in angiogenesis and tumor size (Zhang et al., 2017). PS-targeting liposome, phosphatidylcholine-stearylamine (PC-SA), induced apoptosis and showed potent anticancer effects as a single agent on B16F10 metastatic melanoma mouse xenograft. These liposomes were maximally confined to the tumor and exhibited much less cytotoxicity towards non-cancer cells (De et al., 2018). PS binding peptide (PSBP-6) was conjugated to pH-sensitive (ethylene glycol)-b-poly (D, L-lactide) (PEG-PDLLA) and poly (ethylene glycol)-b-poly (l-histidine) (PEG-PHIS)) micelles. These vesicles were used for encapsulation and delivery of paclitaxel (PTX), a common chemotherapeutic agent (Guan et al., 2020). The peptide-peptoid hybrid PPS1 and the dimeric version of PPS1 (PPS1D1) displayed strong cytotoxicity towards lung cancer cell lines HCC4017 and it’s xenografts in mouse model (Desai et al., 2016). Human A375 melanoma cells that express 50-fold more PS than noncancerous HaCaT cells binds to the A375 melanoma cells with higher affinity for the membranolytic peptide temporin-1CEa (Wang et al., 2016). Similarly, the membranolytic peptide NK2 exhibits preferential interaction with colorectal cancer cell lines (Wilms and Andrä, 2017). Matrix metalloproteinase 2 (MMP2)-sensitive PS-modified nanoparticles were developed to target Tumor-associated macrophages (TAMs) in the tumor microenvironments. The PS exposed on the nanoparticles were recognized by the PS receptors on TAMS that enhanced their apoptotic clearance. This TAM selectivity was successfully reproduced in biological models, including zebra fish, and tumor-bearing mice. The model drug dasatinib was successfully targeted into the TAM cells using the PS nanoparticles (Liu et al., 2020). Liposomes are essential vehicles for targeting lipid soluble compounds such as Zn(II)-bis-dipicolylamine derivatives. DPA-Cy3 [22, 22]/POPC liposomes have preferential binding to MCF-7 breast cancer cells over MCF-12A non-cancer cells due to 3–7 times more PS exposures on MCF-7 (Ayesa et al., 2017). Treatment with an antibody that targets PS (mch1N11) enhanced the anti-tumor efficacy of tumor-directed radio therapy and improved overall survival. The PS expressed on immune cells in TME provides a negative feedback to the tumor cells (Figure 3). However, blocking the PS exposure on surface of immune cells inhibits their contact with the PS receptors that re-polarizes the immune cells into pro inflammatory TAMs and enhances the anti-tumor efficacy of radiotherapy (Budhu et al., 2021)

**TABLE 3:** Targeting the cell surface PS for anticancer therapeutics.

Cell/Tissue	Cancer Cell Type	PS Exposure	Therapeutic Molecules	Effect on Cancer	References
B16F10 mouse xenograft	metastatic melanoma cell	28.5% (in mouse xenograft)	Holthurian glycosamino glycan	Suppressed p38MAPK and ERK1/2 signaling pathways, Reduced tumor by 55%	Zhao et al. (2013)
B16F10 mouse xenograft	metastatic melanoma cell	40%	Stearylamine-Cationic Liposomes	90% reduction in tumor growth	De et al. (2018)
MDA-MBA-231 (2H11) Mouse xenograft	Human breast cancer	2.5 fold of control (Schwann cells)	Fc-Syt1 conjugated monomethyl auristatin E (Bivalent Fc-Syt1_MMAE)	Blocked breast tumor growth	Li et al. (2018)
HeLa cells	Human cervical cancer cell	5% PS	PS-binding peptide-conjugated PEG micelles containing paclitaxel	Up to 80% release of drug, 1.5 to 3 times decrease in IC_50_ for PSBP-6-PEG-PDLLA/PEG-PHIS targeted cells	Guan et al. (2020)
HCC4017 and H460 lung cancer (mice xenografts)	Lung cancer clinical isolate	65–70%	peptide-peptoid hybrid PPS1 and it’s dimer PPS1D1	80% reduction of PPS1D1-Docetaxel treated tumor cells	Desai et al. (2016)
A375 melanoma cells, B16 mouse melanoma cells	Melanoma cells	50-fold more PS exposure compared HaCaT cells	17 aa cationic AMP Temporin-1CEa	Anticancer activity by pore formation and leakage of cellular content	Wang et al. (2016)
Tumor-Associated Macrophages	MMP2-overexpressing tumor site in mice	MMP2-sensitive PS-nanovesicles containing dasatiniba	PSNP-DSB-loaded NPs (pp-PEG-2k linker is cleaved by MMP2 over expressed in TAM cells)	10 fold reduction in 4T1 orthotopic breast tumor	Liu et al. (2020)
MCF-7	Breast cancer cells	3–7 fold PS exposure compared to MCF-12A cells	Zn(II)-bis-dipicolylamine (DPA)-Cy3 [22, 22]/POPC liposomes	3.5 times more cytotoxicity towards MCF-7 cells	Ayesa et al. (2017)
HROC24 HCT116	Patient-derived colorectal cancer cell lines	(5.7 ± 1.9) % (2.2 ± 0.4) % in HROC24 and HCT116 respectively	Anticancer peptide NK-2 and variants	IC_50_ reduced by 2–4 fold	Wilms and Andrä, (2017)
U87-MG GBM)	(Glioblastoma mouse xenografts	High PS exposure	SapC-DOPS, a PS-targeting nanovesicle	3 times reduction of glioblastoma in mice	Blanco et al. (2014); N’Guessan et al. (2020)
MDA-MB-231-luc-D3H2LN	metastatic breast cancer cells (mouse xenografts)	3 fold PS exposure compared to human astrocytes	SapC-DOPS, a PS-targeting nanovesicle	IC_50_ = 25.2 ± 1.5 µM for MDA cells, reduced brain metastasis of breast cancer cells	Blanco et al. (2014)
HUVEC cells	B16F10 (mouse xenograft)	5 fold increase in PS exposure in HUVEC	Annexin V	5–6 times reduction in B16F10 melanoma tumor, reduced angiogenesis	Zhang et al. (2017)
B16	Melanoma tumor (mouse xenograft)	Enhanced PS exposure in TME macrophages	PS targeting antibody (mch1N11)	2 fold reduction in tumor size and 2.5 fold increase in survival time	Budhu et al. (2021)

## Conclusion and Future Prospective

In addition to conventional chemotherapy and radiotherapy, immunotherapy is currently being explored as the most advanced method of cancer therapy. Immunotherapy stimulates immune system of an individual so as to recognize and attack cancer cells. Development of efficient immunotherapeutics involes (i) Enabling the cancer cells to recognize the malignant cell at an early stage (ii) Enabling strong responses against the cancer cells, (iii) Suppressing the anti-tumor signals so as to sensitize the tumor cells towards immunotherapeutics, (iv) Finding novel and universal drug target on malignant cells for efficient immune targeting and (v) designing novel targeting strategies for immune targeting of solid tumors. Both PLSCRs and TMEM16 family members are over expressed in many cancer cell types those are emerging as new targets for anticancer therapeutics. In addition, PS exposed on surface of malignant cells is a universal marker and drug target for immunotherapeutics. Future studies on designing efficient PS-targeting nano-conjugates and development of their targeting strategies will lead to the development of new immunotherapeuics against cancer.

## Glossary

TCGA: The cancer genome atlas
PM: plasma membrane
PL: phospholipid
APTs: Aminophospholipid translocase
MDR: multidrug resistance
PLSCR: Phospholipid scramblase
PS: Phosphatidyl serine
PC: Phosphatidyl choline
SM: sphingomyelin
PE: Phosphatidyl ethanolamine
GPCR: G-protein coupled receptor
Xkr8: XK-related protein8
TMEM16F: transmembrane protein 16F
ANO: Anoctamin
IFN: Interferon
ISG: Interferon stimulated gene
STAT: signal transducer and activator of transcription
EGF: Epidermal growth factor
EGFR: Epidermal growth factor receptor
MK: midkine
TNFR: tumor necrosis factor receptor
ATRA: all-trans retinoic acid
PKCδ: Protein kinase C delta
TME: tumor microenvironment
FITC: Fluorescein isothiocyanate
DC: dendritic cell
NK: natural killer
TAMs: tumor associated macrophages
AML: acute myelogenous leukemia
GCSF: Granulocyte stimulating factor
BCL-2: B-cell lymphoma 2
MCL: mantle cell lymphoma
RA: 9-cis-retinoic acid
CRC: human colorectal cancer
IRS1: insulin receptor substrate 1
TLR: Toll-like receptor
MTA: Methylthioamphetamine
PMA: Phorbol-12 myristate-13 acetate
MVs: Microvesicles
PD1: Programmed cell death protein 1
AREG: amphiregulin
CDK9: cyclin-dependent kinase 9
Mcl-1: myeloid cell leukemia 1
XIAP: X-linked inhibitor of apoptosis protein
SyT1: Synaptotagmin-1
PEG: Polyethylene Glycol
